# Comparative analysis of oral microbiome in molar-incisor-hypomineralization vs healthy age-matched controls

**DOI:** 10.1128/spectrum.02897-24

**Published:** 2025-03-31

**Authors:** Marina Jung, Sébastien Boutin, Marlinde M. Simon, Cornelia Frese

**Affiliations:** 1 Department of Conservative Dentistry, Clinic for Oral, Dental and Maxillofacial Diseases, University Hospital Heidelberg, Heidelberg, Germany; 2 Institute of Medical Microbiology, University of Lübeck and University Hospital Schleswig-Holstein, Lübeck, Germany; 3 German Center for Infection Research (DZIF), Hamburg-Lübeck-Borstel-Riems, Lübeck, Germany; 4 Airway Research Center North (ARCN), German Center for Lung Research (DZL), Lübeck, Germany; University of the Pacific, Arthur A. Dugoni School of Dentistry, San Francisco, California, USA

**Keywords:** oral microbiome, dental plaque, human microbiome, *Streptococcus*

## Abstract

Molar-incisor-hypomineralization (MIH) is one of the most challenging dental diseases in children. While the association of oral microbiomes with caries and periodontitis has been studied thoroughly, limited data on the microbial composition in MIH and its clinical significance exist. This cross-sectional study aimed to compare the supragingival plaque microbiome between children and adolescents affected by MIH and a healthy age-matched control group. Ninety-five patients aged 7–17 years were recruited at the Department of Conservative Dentistry, Heidelberg University Hospital. The final sample included 29 participants with a confirmed diagnosis of MIH, treated preventively and restoratively, and 35 orally healthy controls. Clinical data were obtained, and supragingival plaque samples were collected using OMNIgene ORAL OMR-110 (DNA Genotek Inc.), followed by 16S rRNA amplicon sequencing. The microbiome composition was analyzed using α-diversity (Shannon index) and evenness (Pielou index), with group differences assessed using permutational multivariate analysis of variance (PERMANOVA) and MaAsLin2. The overall microbiome composition showed mostly similarities between both groups (PERMANOVA: *R*² = 0.019, *P*-value = 0.287), indicating no major dysbiosis. However, a significant decrease in α-diversity and evenness was observed with an increasing number of MIH-affected teeth. Pronounced positive correlations were found between ASV0055 (*Streptococcus spp*.), caries experience, and MIH severity. ASV0100 (*Mannheimia sp*.) increased significantly with the increasing number of MIH-affected teeth, whereas ASV0053 (*Bergeyella sp*.) decreased with higher caries experience. In summary, the oral microbiome of children and adolescents with MIH exhibits no significant differences from healthy children and adolescents of the same age group. Depending on MIH severity, the presence of early plaque-forming species and cariogenic biofilm may increase, requiring intensive, tailored preventive care and appropriate restorative treatment to achieve microbial homeostasis.

**IMPORTANCE:**

Molar-incisor-hypomineralization (MIH) represents a significant burden for affected children and adolescents, playing an increasingly important role in pediatric dentistry worldwide. Despite its high global prevalence, data on the microbiome of MIH patients remains limited. This study is the first to compare the oral microbiome composition of MIH patients with a healthy control group, making a significant contribution to pediatric dentistry and microbiology. Our results indicate that the oral microbiome of children with MIH is similar to that of healthy children of the same age. Although this structural anomaly predisposes patients to caries, effective preventive and restorative treatments can help maintain microbial homeostasis. However, MIH-affected children remain high-risk patients, as the disease severity may reduce microbial diversity. Furthermore, the increased abundance of Streptococcus spp. in MIH patients indicates a higher caries susceptibility, emphasizing the need for targeted dental care focusing on plaque control and topical fluoride use.

## INTRODUCTION

Molar-incisor-hypomineralization (MIH) is a challenging dental condition that occurs in children worldwide. It is a developmental enamel defect of systemic origin that affects the first permanent molars and incisors (1). Depending on its severity, MIH can significantly impact children’s dental health and quality of life (2), making research into the causes of this condition particularly important. Its etiology has not yet been fully established. Associations with birth complications and early childhood illnesses have been reported. Recent findings indicate a potential role of genetic and epigenetic factors (3). Nowadays, MIH is believed to be one of the most common non-carious diseases of the tooth structure in children and adolescents (4). Its global prevalence varies from 2.4%–40.2% (5, 6), with recent meta-analyses reporting average rates of 14.2% (7) and 13.5% (8).

Clinically, MIH manifests as demarcated creamy-white to yellow-brownish opacities, with or without posteruptive enamel breakdowns. In severe cases, hypomineralization can lead to such extensive defects that teeth cannot be preserved and, even at a young age, must be extracted (1). The hypomineralized enamel has lower calcium and phosphorus but higher protein content. Therefore, it exhibits increased porosity, significantly reducing the mechanical strength of affected teeth (9). The rough and damaged surfaces lead to plaque accumulation, making daily oral hygiene more challenging, which significantly increases the individual caries risk (10).

Besides caries, tooth hypersensitivity is another characteristic symptom of MIH. It often leads to avoiding certain foods, reduced compliance with maintaining oral hygiene (11), and an increased risk of tooth decay (10). These issues may complicate treatment, requiring a complex approach (12). Therapeutic options for MIH with mild hypersensitivity typically include preventive measures using topical fluoride products (toothpaste, gels, and varnishes) and casein phosphopeptide-amorphous calcium phosphate products. In severe cases, direct and indirect restorations with composites, ceramics, or prefabricated steel crowns, as well as tooth extractions, are indicated (13, 14).

The microbiome and its relevance are gaining increasing interest in research, with oral diseases such as caries and periodontitis already well studied. The oral microbiome is highly diverse, consisting of bacteria, fungi, viruses, archaea, and protozoa, with approximately 700 species inhabiting the oral cavity (15, 16), making it the second-largest microbial community in the human body after the gastrointestinal tract (17). Numerous studies indicate that changes in microbiological homeostasis play a crucial role in the pathogenesis of various oral and systemic diseases (18, 19). Despite its high prevalence worldwide, data on the microbiome of MIH patients remain limited. Until today, only one study has examined the bacterial composition of supragingival plaque in MIH-affected teeth of 25 children (20). While a reduction in bacterial diversity is typically associated with pathological oral conditions (21), Hernandez et al. found that MIH-affected teeth showed a higher microbiome diversity compared to sound teeth. However, this study was conducted in a split-mouth design. Therefore, we aimed to compare the composition of the oral microbiome in children and adolescents with MIH to an age-matched healthy cohort. Furthermore, in the MIH group, associations between clinical parameters—such as caries experience (using the decayed, missing, filled, and teeth index [DMF-T]), MIH severity, number of MIH-affected teeth, gingival health indices (including plaque control record [PCR] and gingival bleeding index [GBI])—and the composition of the oral microbiome were investigated.

## MATERIALS AND METHODS

This cross-sectional study was conducted at the Department of Conservative Dentistry, Heidelberg University Hospital. The study received ethical approval from the local ethics committee (S-550/2021) and was registered with the German Clinical Trials Register (DRKS 00030206). All participants and their legal guardians provided written informed consent in accordance with the Declaration of Helsinki.

A total of 95 patients were recruited between January 2022 and March 2024 in our department, with 45 in the MIH group and 50 in the control group. Clinical examinations and plaque sample collections were carried out during a single visit. The inclusion criteria for both groups included male and female participants aged 7–17 who could maintain regular oral hygiene. To fulfill this criterion, effective plaque control in the home environment, performed by the child themselves or with parental support, had to be ensured. Participants in the MIH group had to be diagnosed with MIH, while the control group consisted of orally healthy participants with sound teeth and no caries experience. Exclusion criteria included the presence of severe or rare diseases, syndromic conditions, or infantile cerebral palsy, as these conditions could impair the ability to perform adequate oral hygiene and affect the composition of the oral microbiome, as well as the intake of antibiotics in the last 2 weeks. Specific dietary habits, individual tooth brushing methods/techniques and duration, or socioeconomic factors were not assessed. Regarding the use of fluoride, the official recommendations for children and adolescents to use fluoride toothpaste (1,450 ppm F^−^ from the age of 6) and a highly concentrated fluoride gel once a week were applied (22).

In the MIH group, 45 individuals were eligible, and 35 agreed to participate, underwent clinical examinations, and had samples collected. In the control group, 50 individuals initially agreed to participate, of whom six were excluded during the oral examination due to the detection of structural defects (*n* = 1), restored teeth (*n* = 3), or active carious lesions (*n* = 2), resulting in 44 participants.

During a comprehensive clinical oral examination by professional dentists, the dental status was documented, including the MIH assessment according to the European Academy of Pediatric Dentistry criteria (23) and severity grading using the MIH treatment need index (MIH-TNI) (24). Tooth sensitivity was evaluated using the Schiff cold air sensitivity scale (SCASS) (25), and caries experience was recorded via the mixed DMF-index (dmft/DMFT; lower-case letters for the deciduous teeth and upper-case letters for the permanent teeth) according to World Health Organization basic methods (26). Additionally, the PCR (27) and GBI (28) were measured in the MIH group.

### Sampling

Sampling of the supragingival plaque was performed using OMNIgene ORAL OMR-110 (DNA Genotek Inc.) at least 1 hour after eating. Participants and their parents/legal guardians were instructed to abstain from brushing their teeth prior to the appointment. The last tooth brushing at home took place the evening before. Using the swab provided in the kit, a small amount of supragingival plaque was collected from the gingival margin of MIH-affected teeth. In the control group, the supragingival plaque was collected analogously in the molar region of the maxilla. The acquired plaque was placed into the collection tube containing stabilizing liquid and stored at room temperature (15°C–25°C) until further processing.

### DNA extraction

DNA extractions were performed using the QIAamp PowerFecal Pro DNA Kit (QIAGEN GmbH, Hilden, Germany). Samples were pretreated with 8 µL Proteinase K (RNA Purification Kit, Lucigen Corp., Middleton, WI) followed by a 15-second vortex and incubation at 50°C for 1 hour. After purification following the manufacturer’s protocol, 50 µL of nuclease-free water was added to the column, and the tubes were left to stand for 5 minutes and centrifuged at 15,000 *g* for 1 minute to elute the DNA.

### 16S rRNA amplicon sequencing

Initially, DNA was amplified using universal bacterial primers targeting the V4 region of the 16S rRNA gene, with both forward and reverse primers being used. Each primer was uniquely barcoded to match the sequences to their respective samples. The PCR mixture was composed of Q5 High-Fidelity 1× Master Mix (New England Biolabs GmbH, Germany) along with 0.5 µM of each primer, 2 µL of DNA, and sterile water, resulting in a 25 µL final volume. Thermal cycling conditions consisted of an initial denaturation step at 94°C for 3 minutes, followed by 30 amplification cycles (94°C for 45 seconds, 50°C for 60 seconds, and 72°C for 90 seconds), and a final extension at 72°C for 10 minutes (Primus 25, Peqlab Biotechnologie GmbH, Germany or FlexCycler2, Analytik Jena AG). Negative controls were performed simultaneously using sterile water as a template to monitor and control contamination. Positive controls from a mock community (HM-782D) were included to assess the PCR and sequencing error rates. The PCR products were checked for amplicon presence through agarose gel electrophoresis (2%) and underwent subsequent purification using Agencourt AMPure XP beads (Beckman Coulter, Germany) according to the manufacturer’s instructions. The quality and concentration of the products were assessed using the Quant-iT PicoGreen dsDNA Assay Kit (ThermoFisher Scientific GmbH, Dreieich, Germany) and the Bioanalyzer (Agilent Technologies Inc., Böblingen, Germany). In the final step, sequencing adapters were ligated to the library, followed by paired-end sequencing on a 250-cycle Illumina MiSeq system. Due to low-quality DNA, some samples did not provide libraries or sequencing failed, resulting in 29 MIH group and 35 control samples.

### Statistical analyses

Data analyses were performed with SPSS, Version 24.0 (29). Descriptive statistics were used to evaluate the characteristics of the study population. The 16S data were analyzed using R3.1.4 with the R package Dada2 (30). The raw sequence data were processed using Dada2 to produce amplicon sequence variants (ASVs) with the following parameters: no ambiguities (*N*) allowed, one error per read allowed, and truncation of the reads at the first position with a quality score of <2. Reads were merged as contigs and checked for chimeras with the default parameters (30). The read counts of each ASV were then used to create an ASV table, and the sequences were classified using the Silva database v138.1. These data were merged into a phyloseq object in R adding the metadata. No negative controls produced reliable amplicons, and the mock community positive control only produced amplicons assigned to the nine expected species, so we did not decontaminate our data set. For each sample, a rarefaction curve was drawn to check that the coverage was correct. The bacterial community was characterized by calculating α-diversity (Shannon index), richness (number of observed ASVs), evenness (Pielou index), and dominance (relative abundance of the most abundant ASV), as well as β-diversity (Morisita-Horn distance) using the microbiome package.

A PERMANOVA was performed to assess the statistical significance of differences between the two sample groups and the influence of various clinical parameters on microbial structure. MaAsLin2 (31) was used for multivariable association modeling to identify differentially abundant species between test and control groups, with group (MIH vs control) and the number ofMIH-affected as fixed effects. Correlations between specific ASVs, the microbiota indexes, and quantitative clinical parameters were evaluated using Spearman’s correlation for ranked, scored, or measured clinical data. The results were graphically summarized in a correlation-based network (Spearman’s ρ > 0.45 or ρ < −0.45, *P*-value adjusted < 0.05). Overall *P*-values were interpreted as purely descriptive and considered significant if they were ≤0.05. We used the Benjamini-Hochberg procedure in order to calculate the false discovery rate and the *P*-value adjusted.

## RESULTS

Study-cohort characteristics and clinical parameters are presented in Table 1. A total of 79 samples were collected from both the MIH and control groups. However, 15 samples could not be amplified because of the insufficient DNA amount after extraction. The final data set comprised 29 participants in the MIH group (mean age 11.3 ± 3.1 years) and 35 in the control group (mean age 10.4 ± 2.1 years). Among the MIH group, the average number of MIH-affected teeth was 6.8 ± 2.9. More than half (62.1%) had a most severe MIH-TNI of 4 (4a, 4b, or 4c), characterized by both hypersensitivity and enamel breakdowns. One-third (34.5%) had the most severe MIH-TNI of 2 (2a, 2b, or 2c), with enamel breakdown but without tooth hypersensitivity. Overall, 47.2% of the affected teeth were restored. Furthermore, the mean GBI and PCR were 2.7% ± 4.0% and 42.6% ± 14.5%, respectively. With special regard to the caries experience, children in the MIH group had an average dmft/DMFT of 4.0 ± 2.4 (Table 1).

**TABLE 1:** Demographic and clinical characteristics of the study population^*a*^

Variable	MIH (*n* = 29)		Controls (*n* = 35)	
	Mean ± SD (range)	*n* (%)	Mean ± SD (range)	*n* (%)
Age (years)	11.3 ± 3.1 (7.3–16.6)		10.4 ± 2.1 (7.0–14.8)	
Sex				
Female		9 (31.0)		15 (42.9)
Male		20 (69.0)		20 (57.1)
DMF index (dmft/DMFT)	4.0 ± 2.4 (0.0–11.0)		0.0 ± 0.0 (0–0)	
GBI (%)	2.7 ± 4.0 (0.0–18.0)		NA	
PCR (%)	42.6 ± 14.5 (23.0–74.0)		NA	
Number of MIH-affected teeth	6.8 ± 2.9 (2.0–12.0)		0.0 ± 0.0 (0–0)	
Number of restorations (of MIH-affected teeth)	3.2 ± 2.3 (0.0–11.0)	93 (47.2)	0.0 ± 0.0 (0–0)	0
Number of extracted teeth (MIH affected)	0.4 ± 1.1 (0.0–4.0)	11 (5.6)	0.0 ± 0.0 (0–0)	0
Most severe SCASS			NA	
SCASS 0		10 (34.5)		
SCASS 1		13 (44.8)		
SCASS 2		4 (13.8)		
SCASS 3		2 (6.9)		
Most severe MIH-TNI				
MIH-TNI 0		0		35 (100)
MIH-TNI 1		1 (3.4)		0
MIH-TNI 2a		2 (6.9)		0
MIH-TNI 2b		3 (10.3)		0
MIH-TNI 2c		5 (17.2)		0
MIH-TNI 3		0		0
MIH-TNI 4a		1 (3.4)		0
MIH-TNI 4b		10 (34.5)		0
MIH-TNI 4c		7 (24.1)		0

^*a*^ Values are depicted in mean, SD, and range. dmft/DMFT, decayed, missing, and filled teeth index (in lower-case letters for the deciduous teeth and in upper-case letters for the permanent teeth); GBI, gingival bleeding index; PCR, plaque control record; SCASS, Schiff cold air sensitivity scale; MIH-TNI, MIH-treatment need index; NA, not applicable.

### Microbiome structure in MIH vs controls

**Fig 1:**
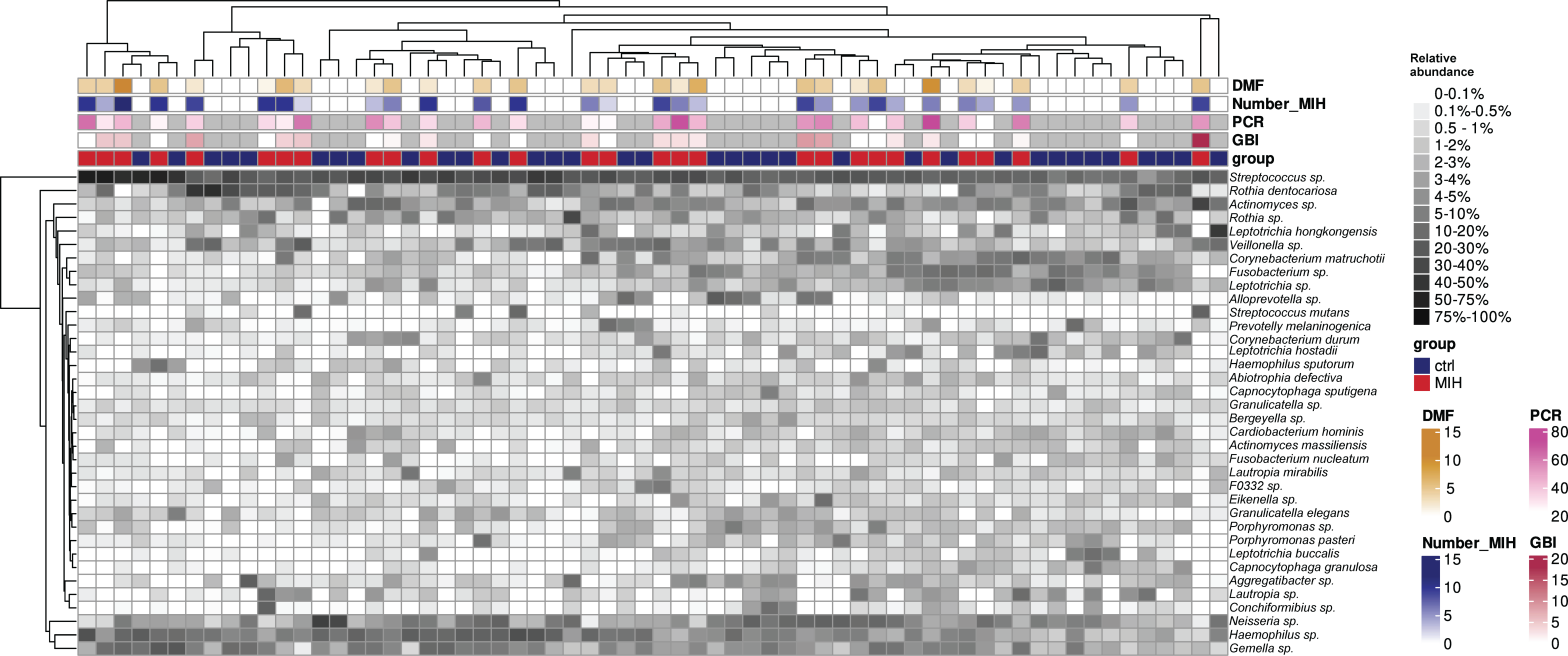
Overview of the microbiome structure at the species level in supragingival plaque samples from MIH (*n* = 29) and control (*n* = 35) groups and its association with clinical parameters. Shown is a two-way clustered heatmap of the 36 most abundant bacterial species (rows). The dendrograms at the top and left display the hierarchical clustering of samples and bacterial taxa, respectively. Shorter branch heights indicate greater similarity between clusters, revealing an overlap in the microbial structure between MIH and control groups. The color key and relevant clinical parameters (indicated by color codes) are presented on the right, showing caries experience by means of the DMF-T index, the number of MIH-affected teeth (Number_MIH), the plaque index (PCR), and the bleeding index (GBI). Clinical parameters presented in the figure are detailed in Table 1.

**Fig 2:**
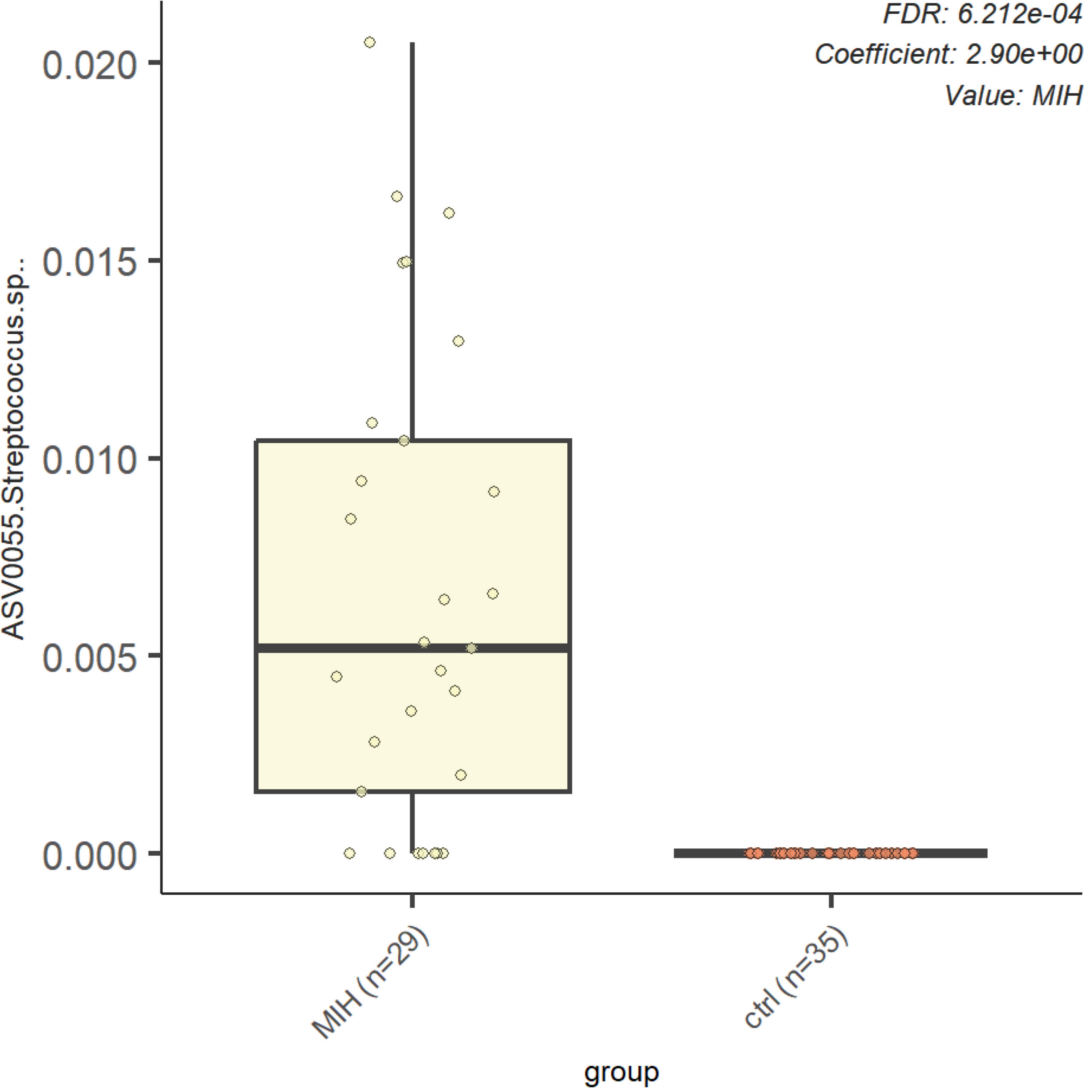
Relative abundance of *Streptococcus sp*. in the MIH and control groups. Bar plots showing the difference in relative abundance (Y-axis) of *Streptococcus sp*. between the MIH and control group (X-axis).

The overall microbiome composition showed mostly similarities between the MIH group and the control group (PERMANOVA: *R*² =0.019, *P*-value = 0.287) indicating no major dysbiosis. In total, 36 bacterial species were observed with a mean relative abundance >0.1% in the whole population (Fig. 1). The *Streptococcus spp*. were most prevalent and abundant in the MIH group. When performing MaAsLin2 analysis, we observed a significant increase in the relative abundance of one specific ASV0055, belonging to *Streptococcus spp*., in the MIH group compared to the control group (*P* < 0.0001; Fig. 2). Furthermore, there was no significant difference between the MIH and control group in the relative abundance of *Actinomyces*, *Corynebacterium*, *Rothia*, *Veillonella*, and *Centipeda* (Fig. S1).

**Fig 3:**
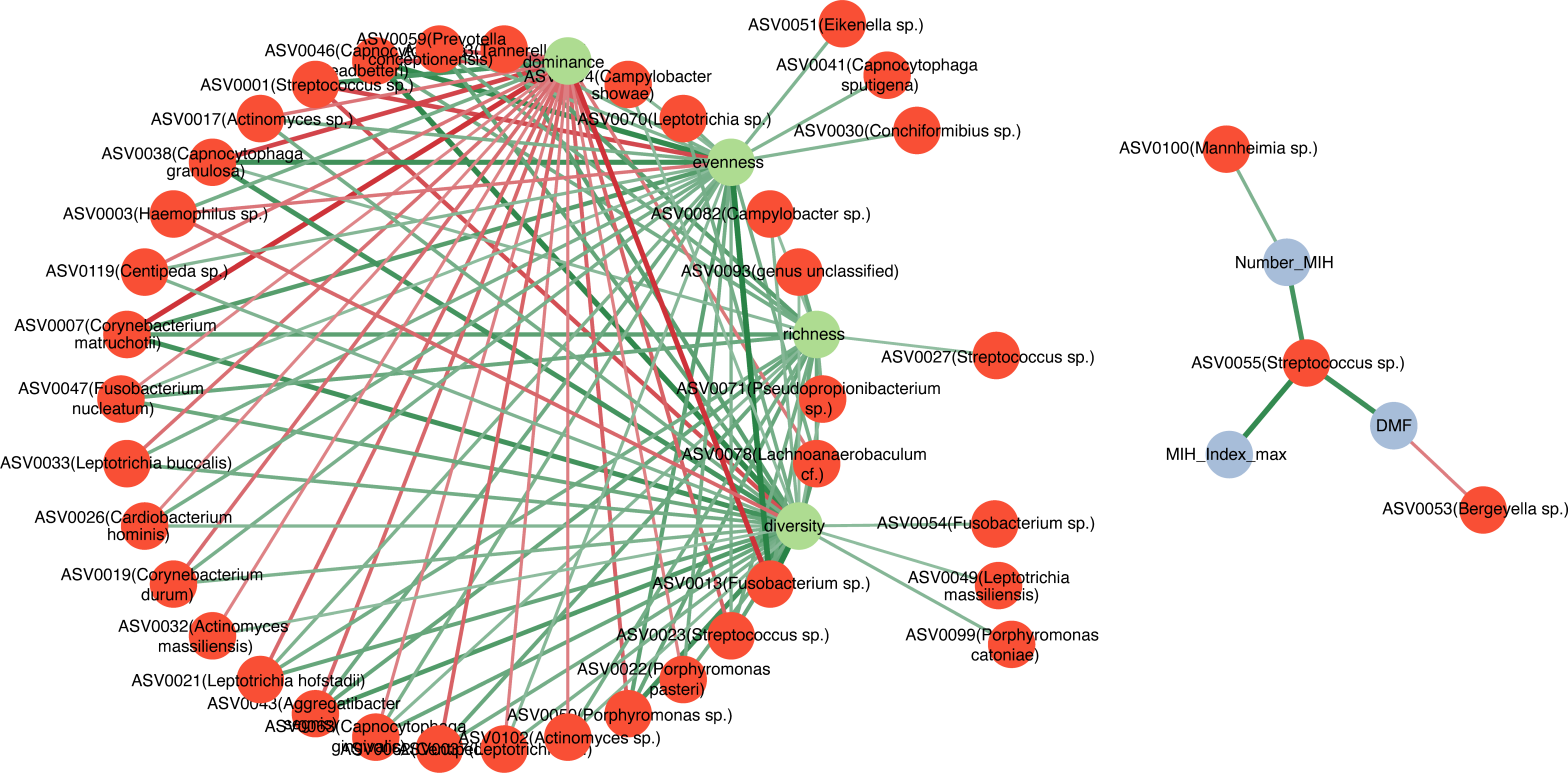
Correlation-based network visualizing pairwise correlations between specific ASVs, α-diversity index, and clinical parameters. Each edge stands for a considerable (Spearman’s ρ > 0.45or ρ < −0.45) and significant (*P* < 0.05) correlation. The different colors indicate positive (green) and negative (red) correlations. The size of the edge indicates the ρ-value; the bigger the edge, the stronger the association.

In the correlation-based network between specific ASVs, α-diversity index, and clinical parameters, we a observed significant correlation between taxa and α-diversity, which was mostly positively oriented (Fig. 3). It indicates a strongly interconnected community linked to higher plaque diversity, while ASV0001 (*Streptococcus sp*.) and ASV0003 (*Haemophilus sp*.) were associated with a more single-dominated microbiome. Only a few taxa were correlated with clinical parameters: we observed positive correlations between ASV0055 (*Streptococcus spp*.) and caries experience (ρ = 0.74, *P*-value < 0.001) and MIH-TNI (ρ = 0.78, *P*-value < 0.001) and the number of MIH-affected teeth (ρ = 0.70, *P*-value < 0.001). ASV0100 (*Mannheimia sp*.) increased significantly with a higher number of MIH-affected teeth (ρ = 0.47, *P*-value = 0.020), whereas ASV0053 (*Bergeyella sp*.) decreased with increased caries experience (ρ = −0.45, *P*-value = 0.030; Fig. 3; Fig. S2).

### Bacterial diversity

**Fig 4:**
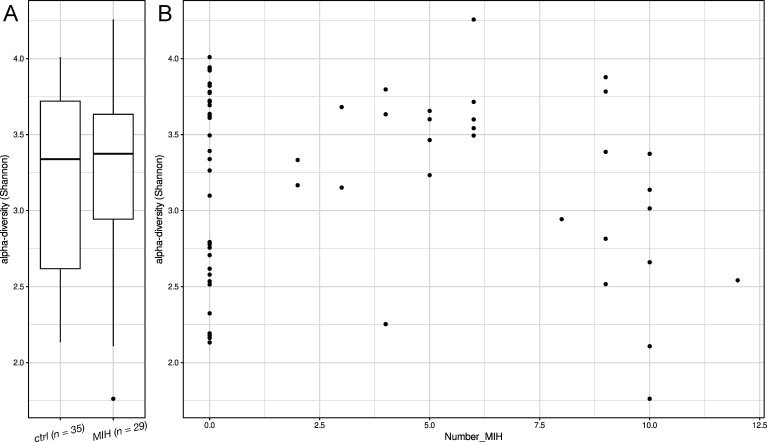
Bacterial diversity. (A) The boxplot of the Shannon index presents the differences in α-diversity between the MIH (*n* = 29) and control groups (*n* = 35). (B) α-Diversity based on the Shannon index (Y-axis) in correlation with the number of MIH-affected teeth (Number_MIH; X-axis) assessed for the MIH and control groups (Number_MIH = 0).

There was no significant difference in the α-diversity (Shannon index) in the whole population of MIH patients and controls (Fig. 4A). Figure 4B shows the α-diversity for both MIH and control groups compared to the number of MIH-affected teeth, with the healthy controls all on the zero line. In MIH subgroup analysis, we observed a significant association between the α-diversity (ρ = −0.42, *P*-value = 0.024) and evenness (ρ = −0.47, *P*-value = 0.010), as with an increasing number of MIH-affected teeth, both decreased significantly. The correlations between α-diversity and all other clinical parameters, such as GBI, PCR, dmft/DMFT, most severe SCASS, most severe MIH-TNI, and the number of restored and extracted teeth, were not significant.

## DISCUSSION

The objective of the study was to compare the supragingival plaque microbiome between MIH-affected and healthy age-matched children and adolescents. To the best of our knowledge, this study is the first to investigate the composition of the oral microbiome in MIH patients compared to a control group of orally healthy peers. Yet, only a limited number of studies have investigated the oral microbiome in MIH patients to date, making our study particularly noteworthy.

Our findings indicate that the overall microbiome composition was highly similar between children and adolescents with MIH and those with healthy oral conditions. This suggests that the composition of supragingival plaque in MIH patients maintains a relatively normal profile (Fig. 1). In this study, MIH patients affected by caries due to their structural anomaly had already received restorative treatment; therefore, there was no interaction with a dysbiotic, caries-associated microbiome. This factor is of crucial importance for the interpretation of the results, as it ensures that the differences in the microbiome structure are not superimposed by caries-related dysbiosis and can be attributed exclusively to the non-carious aspect of MIH. The therapeutic consequence of treating MIH patients should generally be directed to create or sustain this microbial homeostasis. The similarities in the oral microbiomes of both groups indirectly indicate that children with MIH and previous caries experience (dmft/DMFT of 4.0 ± 2.4; Table 1) were able to return to a homeostatic state after preventive and restorative therapy.

However, we observed a significantly higher abundance of a specific ASV belonging to the *Streptococcus* species in the MIH group (Fig. 2). *Streptococcus* species are common inhabitants of the oral cavity (32). As a part of the early colonizer complex, they influence plaque formation and development (33). While *Streptococcus sanguinis* is associated with healthy oral conditions (34), *Streptococcus mutans* is linked to dental caries being particularly frequent in the oral cavity in individuals with caries (35, 36). Clinically, a significantly higher abundance of *Streptococcus spp*. in the oral microbiome is related to an increased tendency for plaque formation on dental surfaces. This is favored by the rough and uneven surfaces of MIH-affected teeth and may explain the increased presence of *Streptococcus spp*. Additionally, more severe forms of MIH show extended defects, providing an ideal environment for bacterial colonization due to the higher porosity. Severe MIH (MIH-TNI 4a–4c) is also associated with tooth hypersensitivity, negatively impacting the oral hygiene of affected children and adolescents (24). Patients experiencing sensitivity or even pain are more likely to brush their teeth less thoroughly, which leads to further plaque accumulation. Our results indicate that although the oral microbiome in MIH patients largely retains its typical structure, the increased presence of *Streptococcus spp*. may explain the higher susceptibility of MIH-affected teeth to caries observed in previous clinical studies (10). In order to obtain more precise results at the species level, metagenomic analyses would be required, which are technically much more complex and cost intensive. Consent for such analyses on the *ex vivo* biofilm samples from this cohort was not granted by the ethics committee and thus the parents/guardians.

Pappa et al. reported a lower microbial diversity in MIH patients compared to a healthy control group based on a bacterial proteome analysis of saliva (37). A highly diverse microbiome seems to be stable, while reduced microbial diversity is linked to dysbiosis and, thus, pathological conditions (21). In our study, only the MIH subgroup analysis showed a significant decrease in α-diversity and evenness with an increasing number of MIH-affected teeth. In general, however, there was no significant difference in α-diversity between the MIH and control groups. This finding is likely due to the high heterogeneity within the control group, resulting in a wide range of microbial diversity. This variability can be explained by several factors influencing the oral microbiome in children and adolescents, such as lifestyle (38), dietary habits (18), or oral hygiene routine (18), and could mask potential differences between groups. The increased presence of *Streptococcus spp*. in the supragingival plaque of MIH patients demonstrated in our study supports the theory that it may attach more effectively to the porous surfaces of MIH-affected teeth, displacing other less abundant species and potentially reducing microbial diversity. With an increasing number of MIH-affected teeth, this effect might further aggravate, possibly leading to dysbiosis.

In contrast, Hernandez et al. observed generally higher diversity on hypomineralized teeth, suggesting that enamel degradation increases surface porosity, leading to higher bacterial attachment and diversity (20). Their study was performed in a split-mouth design, comparing plaque samples of MIH-affected and sound teeth within the same patient, without including a control group. Moreover, only MIH-affected teeth without enamel breakdown and atypical restorations were included, and caries experience based on the dmft/DMFT was not reported. Therefore, it remains unclear whether the children had active caries potentially affecting microbial diversity. Nevertheless, Hernandez et al. observed a higher abundance of several proteolytic microorganisms on the hypomineralized teeth. Although streptococci are primarily saccharolytic (39), they also show proteolytic activity, utilizing salivary proteins as a nutrient source to colonize areas of the oral cavity (40). It would be interesting to determine whether the specific proteins found in the enamel of MIH-affected teeth could promote the proteolytic activity of streptococci, which could favor the bacterial penetration of hypomineralized teeth, as also suggested by Hernandez et al. for the proteolytic bacteria they found.

Although there are only three studies on the oral microbial composition of children with MIH to date, their biofilms show a tendency toward species of early plaque formation and potential cariogenic pathogenicity. The therapeutic approach should, therefore, focus on establishing and maintaining microbial homeostasis. This should comprise targeted preventive care, including sufficient chemical and mechanical plaque control. The regular application of sufficiently high-concentrated topical fluorides is of crucial therapeutic importance in the clinical management of MIH. Studies by Pandit et al. have shown that the cariogenic *Streptococcus mutans*, in particular, can be inhibited in its acid production, acid tolerance, and extracellular polysaccharides (EPS) formation by fluorides in a dose-dependent manner (41). However, the effect of a 1-minute fluoride application on acidogenicity, aciduricity, and EPS formation only occurs at a minimum concentration of 300 ppm F^−^ and follows a concentration-dependent manner up to 2,000 ppm F^−^ (42). Based on our results, it can be concluded that brief exposure to a fluoride product with a sufficiently high concentration is an effective preventive measure for MIH and can contribute to maintaining a healthy microbial balance in the oral cavity. Furthermore, it is essential to consider treatment approaches that explicitly address the hypersensitivity of MIH-affected teeth to enhance the patient’s ability to perform effective mechanical plaque control without any pain-related limitations. Along with fluoride products, fissure sealants have proven effective in reducing dental hypersensitivity (43) and are considered the first choice for preventing caries and post-eruptive enamel breakdown in MIH-affected teeth (44, 45). Regarding more invasive treatment options, it may be beneficial to remove the affected porous enamel completely through restorative therapy to reduce the surface available for adhesion and penetration of *Streptococcus* and other bacterial species. In addition to minimizing plaque accumulation sites, removing structurally compromised enamel also improves the longevity of restorations, as demonstrated in clinical studies (46–48).

Although our study has shown many unprecedented results, it has some limitations, including the monocentric cross-sectional setting with limited cohort size and the inability to include the full calculated sample size. Due to these methodological factors, no causalities can be demonstrated. Therefore, we believe that intra-oral conditions such as rough tooth surfaces and often limited oral hygiene due to hypersensitivity promote the increased abundance of *Streptococcus* species in particular. In addition, other factors that may affect the oral microbiome, such as dietary habits, lifestyle, and other environmental variables, were not recorded. This may have contributed to the high heterogeneity observed within the control group, even though all patients were healthy and free of oral diseases. The lifestyle and dietary habits of children and adolescents are not comparable to those of adult cohorts, which may be one reason for the limited data available in the current literature. Therefore, prospective studies with larger sample sizes should take these factors into consideration in the future.

### Conclusions

In summary, our findings highlight that the microbiome of children with MIH does not differ significantly from that of healthy children of the same age. Although the structural anomaly favors carious lesions, microbial homeostasis can be achieved if these are well treated preventively and restoratively. However, children affected by MIH remain high-risk patients, as the severity of the disease and the number of MIH-affected teeth might impair microbial diversity. Furthermore, the increased abundance of *Streptococcus spp*. in MIH patients indicates a higher caries susceptibility, emphasizing the need for targeted dental care focusing on plaque control and the use of topical fluoride. Further studies with larger cohort sizes and metagenomic approaches are needed to evaluate the possible role of the proteolytic activity of the microorganisms in the supragingival plaque of MIH patients and their effects on dental hard tissues.

## Data Availability

Data are available in the SRA database under the Bioproject PRJNA1224592 (49).

## SUPPLEMENTAL MATERIAL

Supplemental material spectrum.02897-24-s0001.docx Fig. S1 and S2.

ASM does not own the copyrights to Supplemental Material that may be linked to, or accessed through, an article. The authors have granted ASM a non-exclusive, world-wide license to publish the Supplemental Material files. Please contact the corresponding author directly for reuse.
