# Supplementary material for: Comparative analysis of oral microbiome in molar-incisor-hypomineralization vs healthy age-matched controls
Source: Microbiol Spectr. 2025 Mar 31;13(5):e02897-24. doi: 10.1128/spectrum.02897-24 (PMC12054143; doi:10.1128/spectrum.02897-24)

**Supplementary Materials**

eFigure 1. Differentially Abundant Bacterial Taxa in the MIH and control groups.

eFigure 2. Correlation between differentially abundant bacterial taxa and number of MIH-affected teeth.

**eFigure 1. Differentially Abundant Bacterial Taxa in the MIH and control groups.**


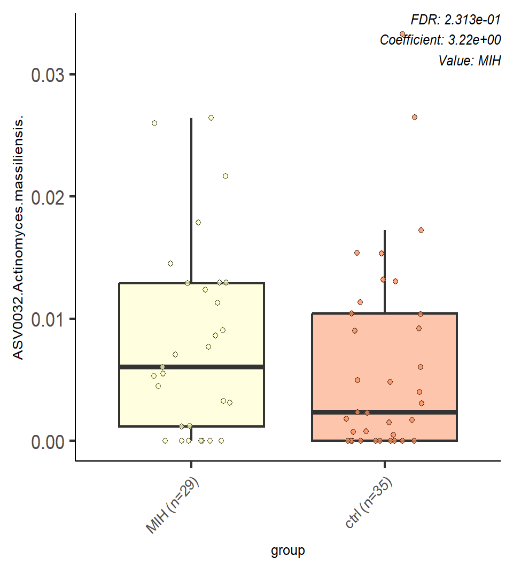

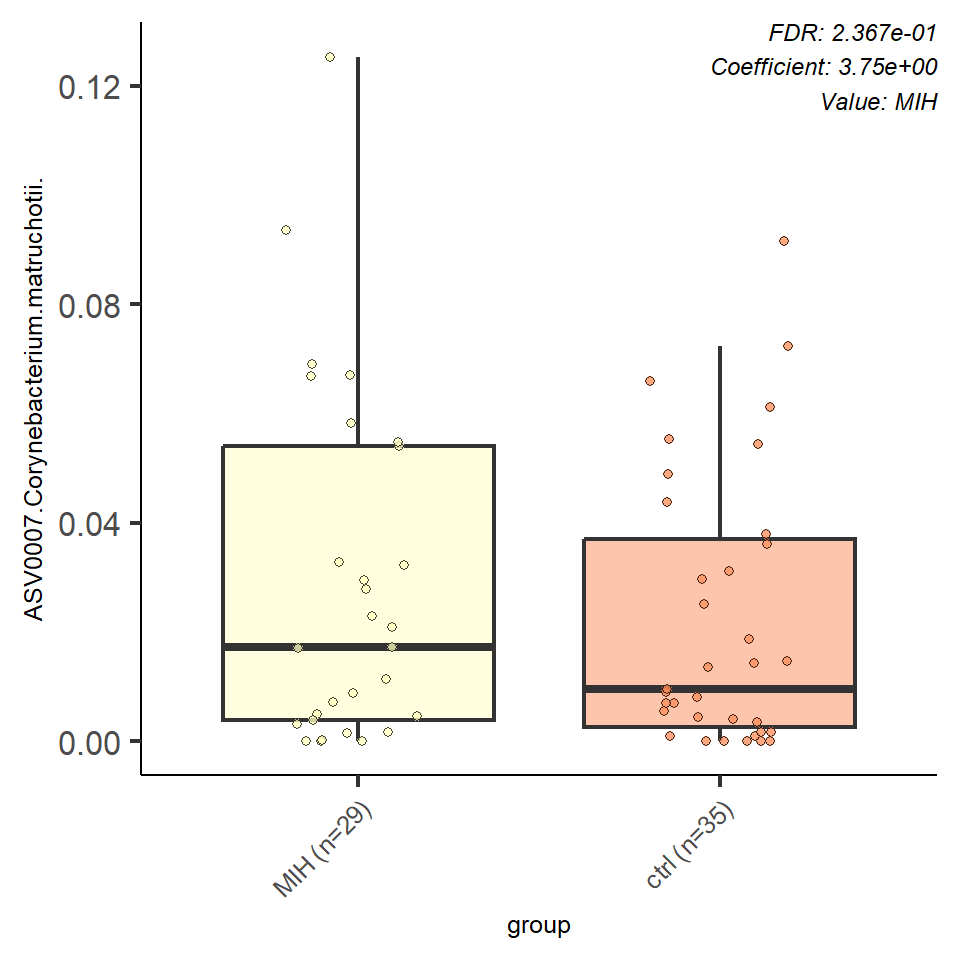

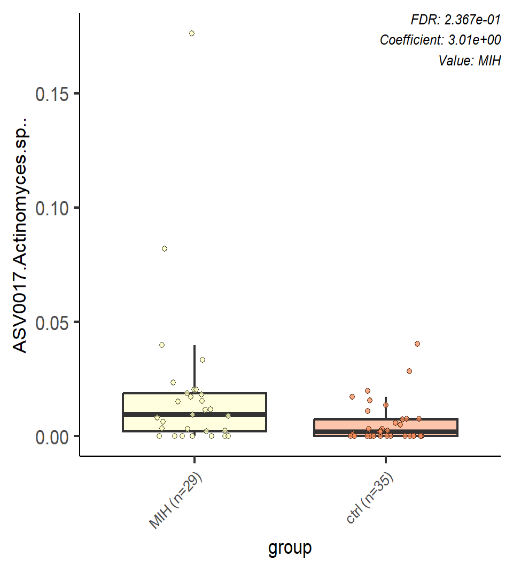

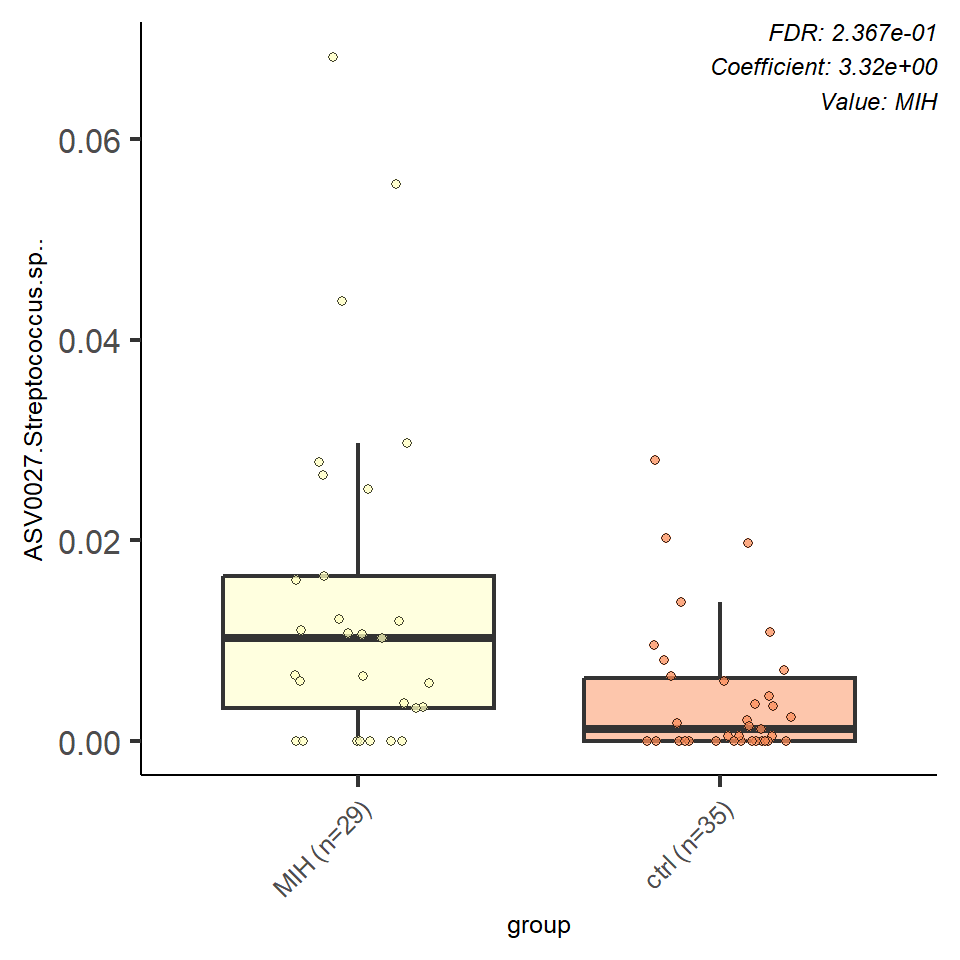

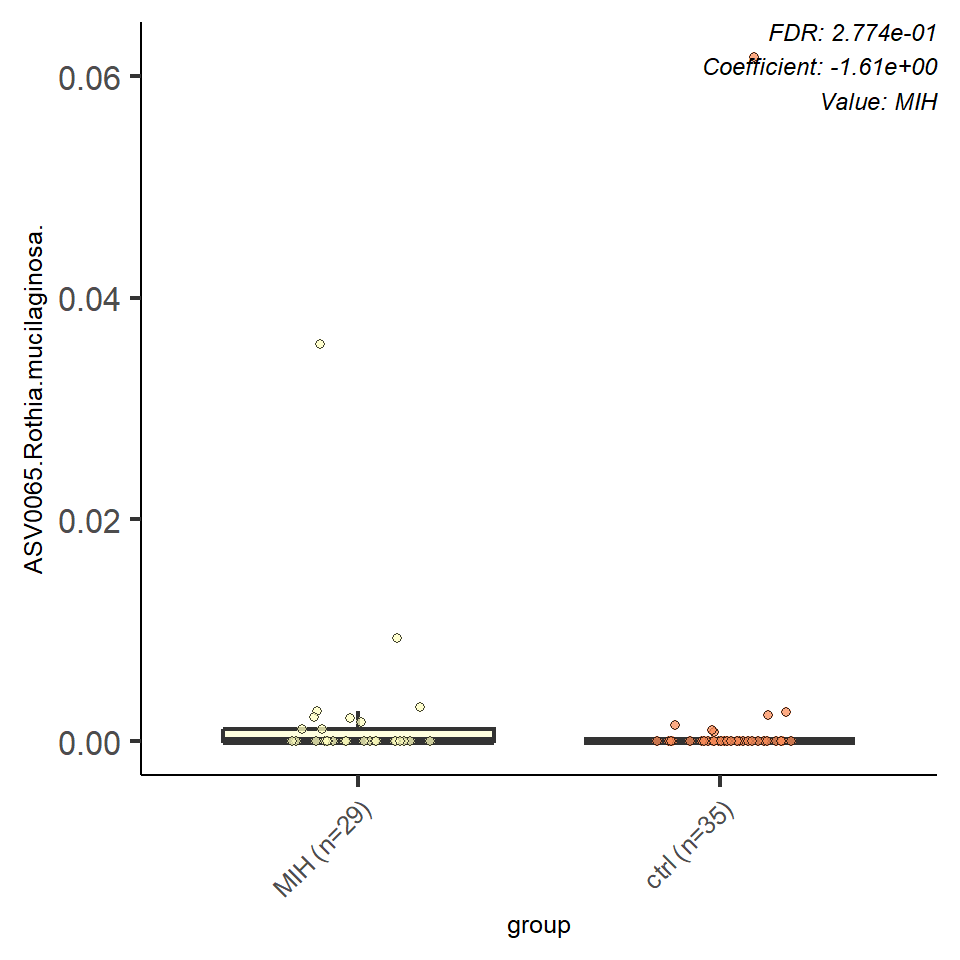

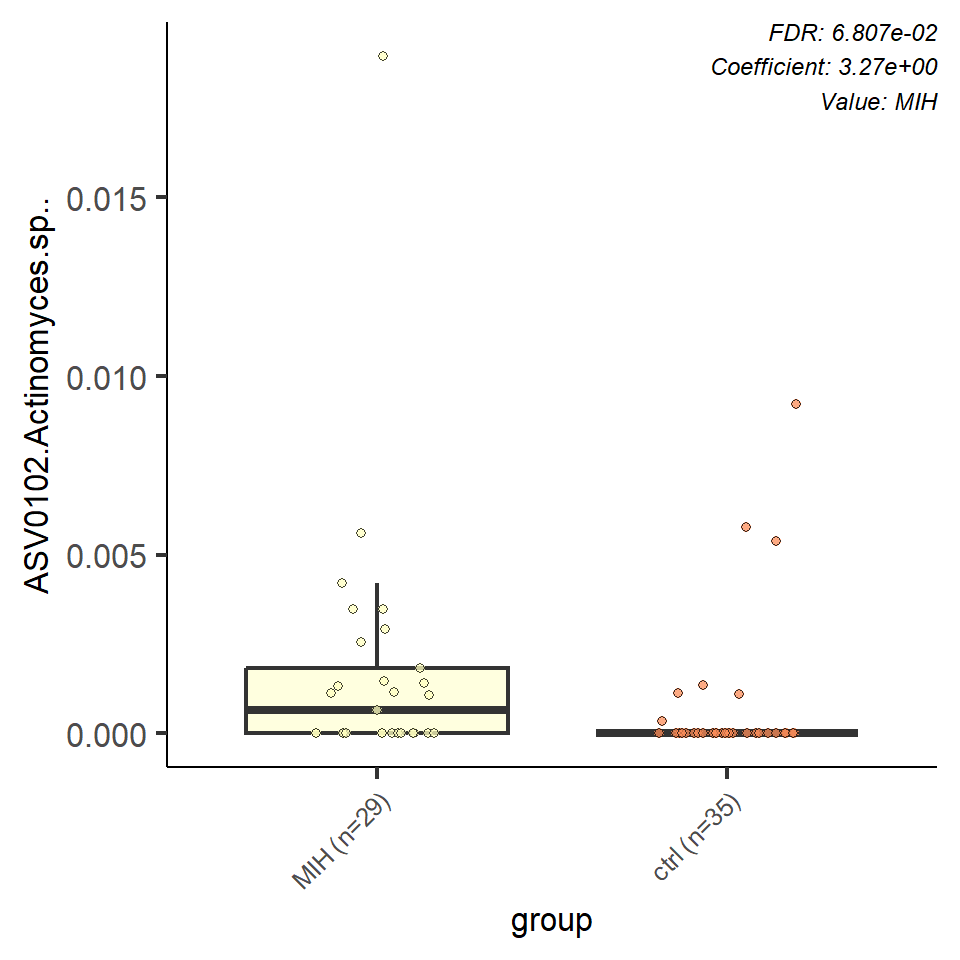

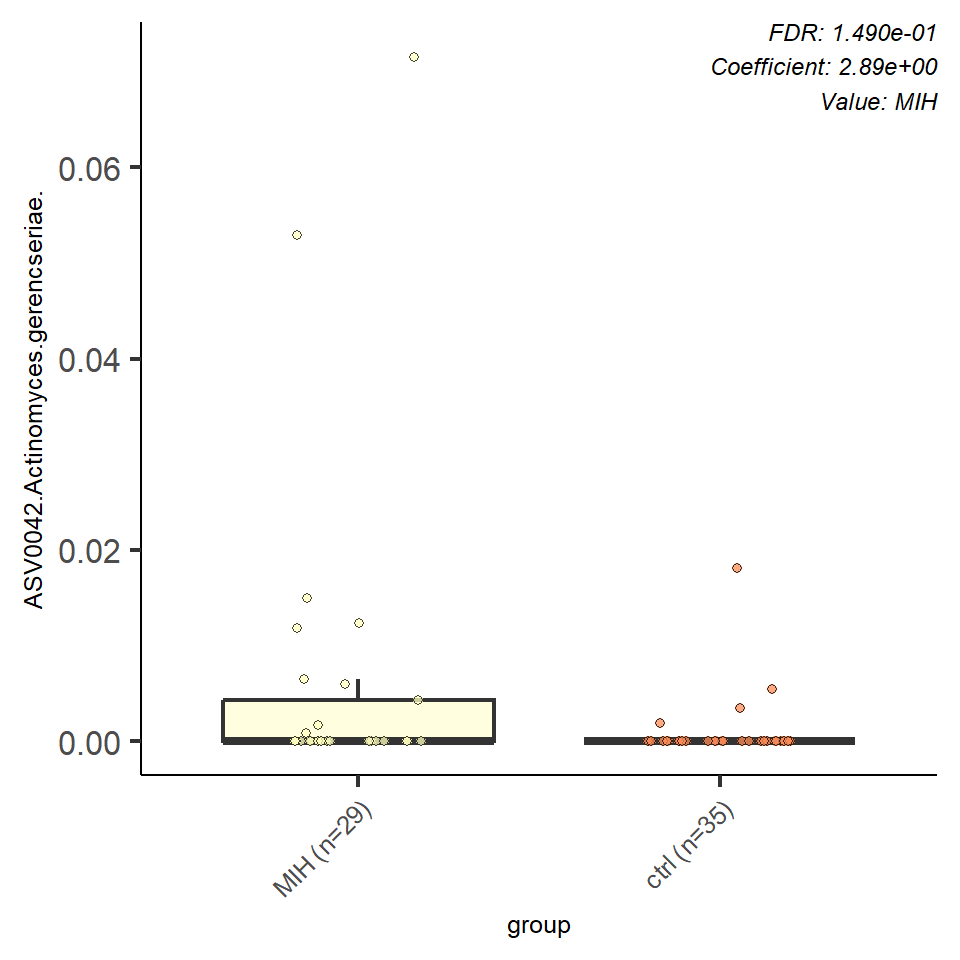

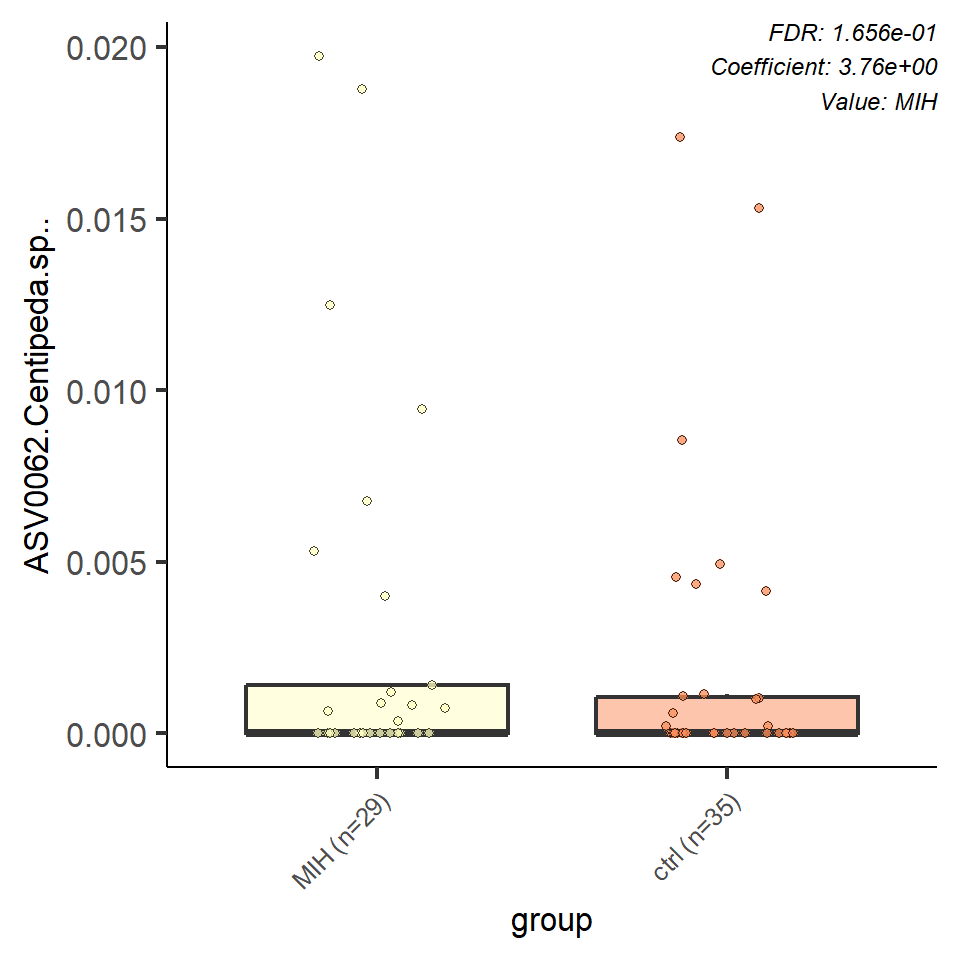

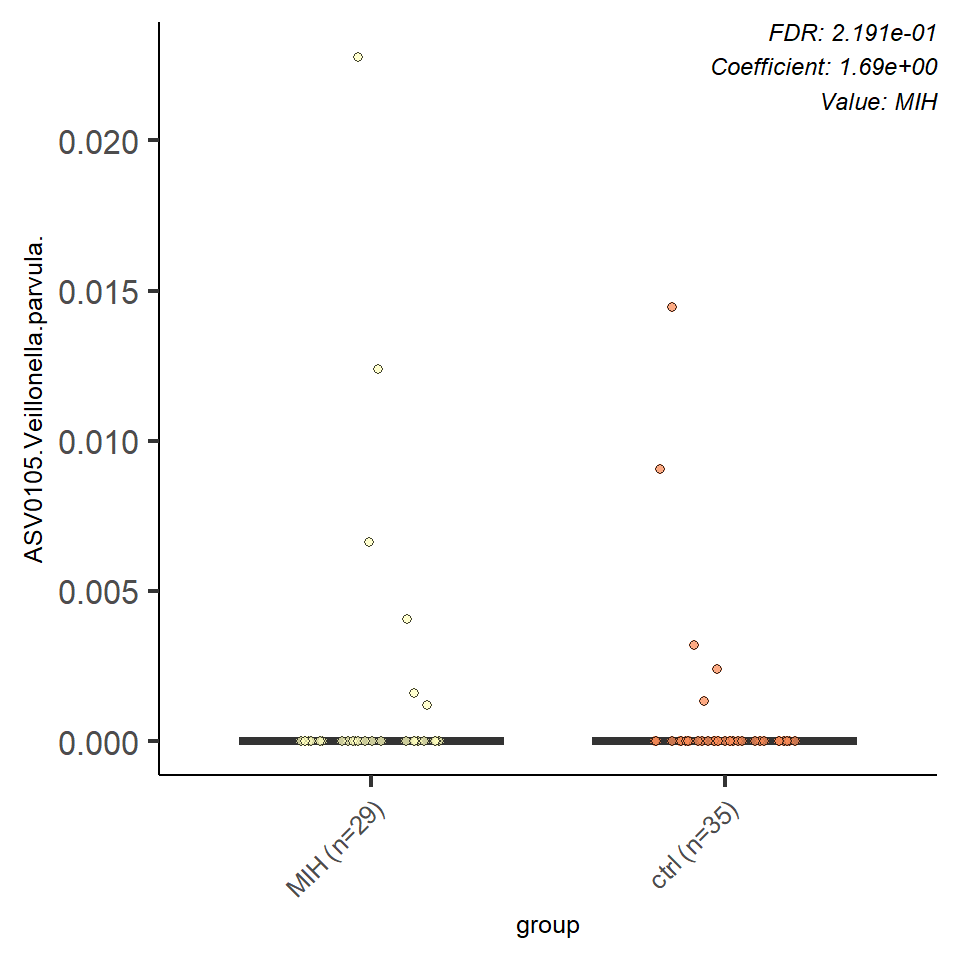


Bar plots showing the difference in relative abundance (Y-axis) of bacterial taxa between the MIH and control group (X-axis).

**eFigure 2.** **Correlation between differentially abundant bacterial taxa and number of MIH-affected teeth.**

Linear mixed models implemented in MaAsLin2 were used to analyze the correlations. The Y-axis represents the relative abundance of the respective bacterial taxa, while the number of MIH-affected teeth (Number_MIH) is presented on the X-axis.


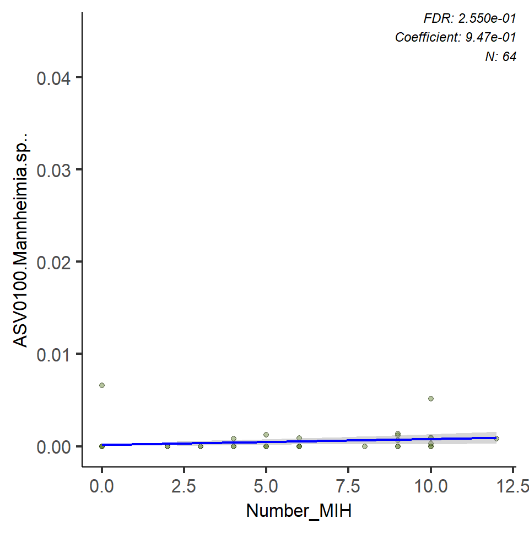

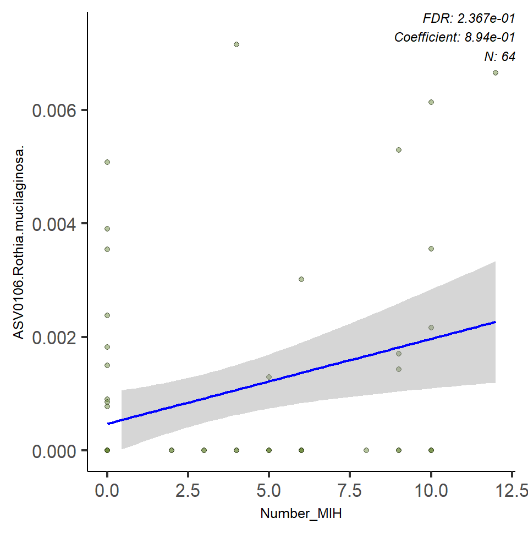

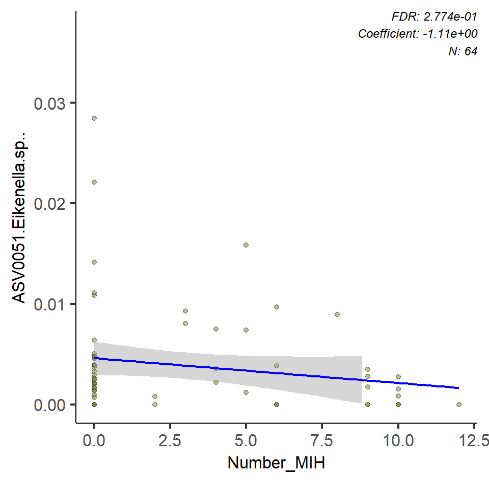

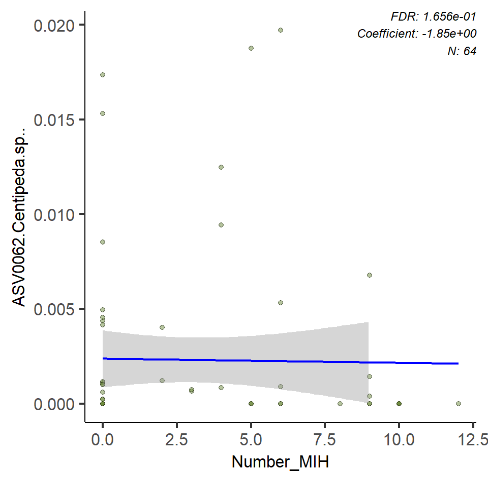

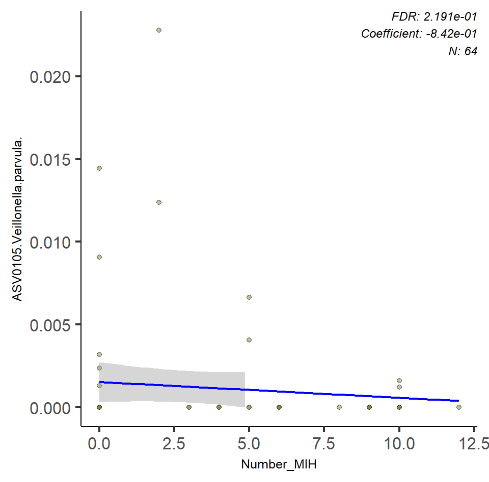

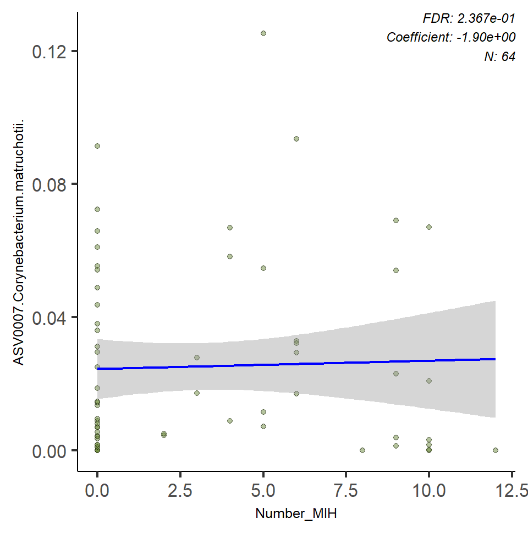

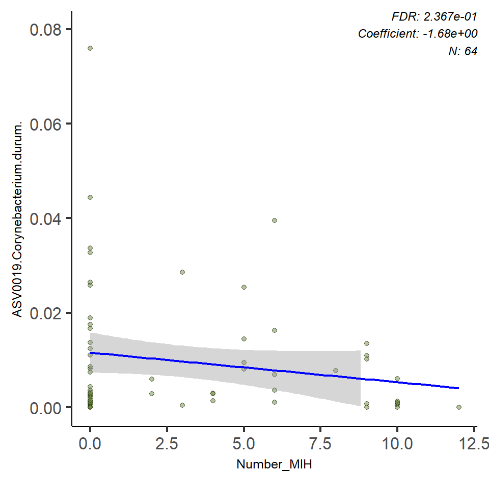

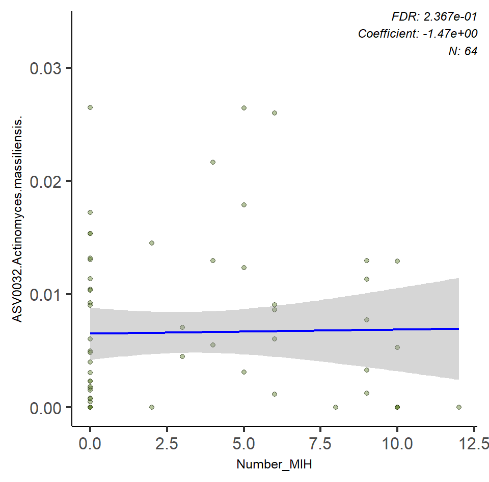

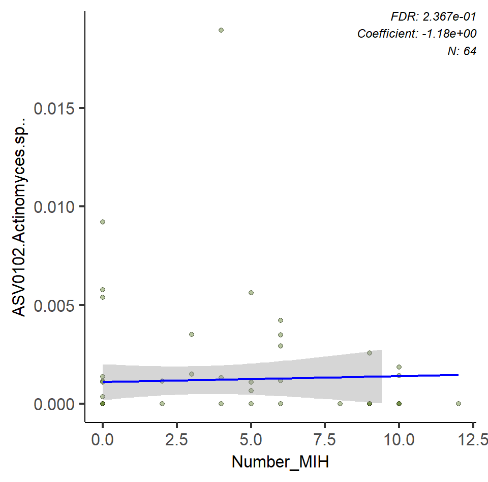

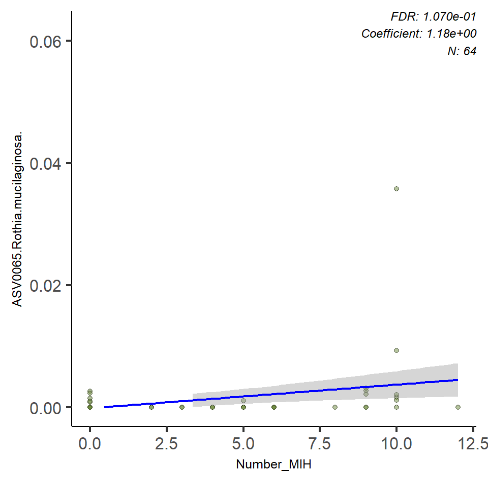

Supplement: Supplemental material — Fig. S1 and S2. [file spectrum.02897-24-s0001.docx]
